# Enhancing pathology learning for medical students – via blended learning by clinicians

**DOI:** 10.15694/mep.2021.000082.1

**Published:** 2021-03-29

**Authors:** Sankar Sinha

**Affiliations:** 1 University of Tasmanian and University of Notre Dame Australia

**Keywords:** Blended learning, TPACK, pathology teaching supplement, medical education, critical thinking and clinical reasoning.

## Abstract

This article was migrated. The article was marked as recommended.

**Objectives:** Assessing the feasibility and benefits of supplementary pathology teaching by a clinician to improve students’ understanding of the pathological process and its relationship with clinical symptoms and signs during their clinical rotations.

**Design, Setting, and Participants:** The Bachelor of Medicine and Bachelor of Surgery (MBBS) Course in the University of Tasmania is a 5-year undergraduate program and all disciplines of pathology are taught in years 1, 2 (pre-clinical) and 3 (clinical transition), by pathologists. Over a twelve-year period, with the application of technology, pedagogy, and content knowledge framework, a blended pathology learning program developed and delivered by a surgeon to supplement the existing pathology curriculum. Participants were medical students in year three of the MBBS course during their nine-week surgical rotations.

**Intervention:** Images of pathology specimens were presented online for the students to identify the organ, pathology and associate this with clinical features to arrive at a clinical diagnosis using online team discussions followed by face-to-face sessions to further explore students’ responses.

**Main outcome measures:** The survey used both quantitative and qualitative measures to assess students’ knowledge gain in pathology, their perceptions about critical thinking and clinical reasoning skills, and satisfaction.

**Results:**The results indicate that most students’, especially the weaker students, improved on their pathology knowledge over the period of the course, which helped in their development of skills in diagnosing and managing diseases with a high level of satisfaction of all students.

**Conclusion:** This study provides supportive evidence that overall students’ pathology knowledge improved through delivery using blended learning and importantly weaker students benefitted considerably. Also, the results suggest that when students view pathology specimens online, engage in scholarly debate about them, and participate in face-to-face sessions to consolidate their learning, they gain strong inductive medical reasoning skills.

## Introduction

In medicine, pathology is the bridge between basic sciences and clinical practice ( Marshall, Cartwright and Mattick, 2004). A sound grasp of pathology is required in all areas of clinical practice, requiring an accurate diagnosis, planning of treatment, and estimating prognosis. Regrettably, pathology teaching in medical schools has decreased in recent years ( Domizio and Wilkinson, 2006 and Mortimer and Lakhani, 2008) driven by changes in the delivery of medical education and the many divergent opinions. The importance of pathology as a subject in medical education has ranged from being not essential to being central to the understanding of disease - which is an important process of becoming a good physician ( Burton, 2005; Hermann, *et al.,* 2015).

Reasons for the reduction of pathology in the medical curriculum are the introduction of integrated curricula in the form of problem-based learning (PBL) ( Mortimer and Lakhani, 2008); a worldwide decline of autopsy rates ( Mortimer and Lakhani, 2008 and Bamber *et al.,* 2014); reductions in the learning opportunity afforded by potted specimens from pathology museums; and resource limitations leading to ‘insufficient or inadequate contact with pathology or pathologists in medical school’ ( Ford, 2010). Remedial approaches to the teaching of pathology in the medical curriculum have been described ( Boon, Smallman, Thompson, 1988; Fenderson, 2005; Lam, Veich and Hays, 2005; Maley *et al.,* 2008; Diaz-Perez, Raju, Echeverri, 2014; Murthy, 2014; Nautiyal *et al.,* 2018), but evidence supporting any of these approaches as best practice is limited. In the UK a study ( Marsdin and Biswas, 2013) was conducted of junior doctors to find out how well their undergraduate pathology teaching prepared them for clinical work. The junior doctors reported that although pathology was a significant component of their postgraduate examinations, their undergraduate courses had not prepared them well for their exams.

In clinical practice, medical students need to learn critical thinking and clinical reasoning skills. Studying pathology and using diseased organs from the pathology museum (known as ‘pots’) can help students to learn critical thinking and clinical reasoning skills. This is through the appreciation of the pathological basis of clinical symptoms and signs in patients, reinforcing situations observed during their clinical rotations.

The contributions of the surgical fraternity in recent years have led to the resurgence of anatomy teaching for undergraduate medical students. As a general surgeon, the author has developed a blended learning program, which has been implemented and refined at the Hobart Clinical School of the University of Tasmania over the past eleven years. The blended learning program now formally adopted is a combination of visual stimulus, online discussion, with its innovative question and answer format, followed by face-to-face tutorial-style discussion. Of interest is that during the current COVID-19 pandemic and face-to-face sessions being cancelled the course format allowed a seamless transition to being delivered by online virtual live tutorials.

In the development of this course, the approach was a practical one and is intended to supplement the existing pathology curriculum without overloading the already-stretched, time-poor, and diminishing number of academic pathologists within the medical school. The main premise of this study is that students will develop a better understanding of the pathological process and its relationship with clinical symptoms and signs through supplementary pathology teaching by a clinician (in this program by a surgeon) during their clinical rotations.

To put it simply, the research questions in this study are:

1. Does a blended learning model with online images from the pathology museum followed by face-to-face sessions improve students’ pathology learning?
2. Do the students develop better insight into the pathological process and reasoning skills to apply them in understanding the resulting clinical symptoms and signs?

## Methods

### 2.1 Context

The Bachelor of Medicine and Bachelor of Surgery (MBBS) Course in the University of Tasmania is a 5-year undergraduate program and is discipline-based, arranged by organ systems, and integrated with clinical practice through case-based learning (CBL). All disciplines of pathology are taught in years 1, 2 (pre-clinical), and 3 (clinical transition), by pathologists/pathology registrars. Pathology is assessed in written short answer questions, multiple-choice questions, and applied questions where students are presented with a clinical scenario with pathology results, potted specimen, and/or microscopic slides.

This new program was progressively developed from 2008 as an adjunct to the existing pathology curriculum as mentioned above. The approach of developing the online module resembled the guidelines by Minasian-Batmanian (2008), with five stages of developmental cycles consisting of analysis, design, development, testing, and evaluation. In each stage, there are phases of planning, acting, observing, and reflecting leading to an iterative process of development. The following description is the final iteration of the blended learning of surgical pathology based on the technology, pedagogy, and content knowledge framework (TPACK) ( Koehler and Mishra, 2008).

The blended learning module ( Garrison and Kanuka, 2004) was developed for students in their 3 ^rd^ “clinical transition” year, who were undertaking their 9-week surgical rotation. Histopathology was not included in this program as this is being dealt with adequately within the existing pathology curriculum. The emphasis on clinical correlation of the macroscopic appearance of potted specimens by the students, making the transition from largely preclinical to clinical teaching, and facilitated by a clinician, was deemed to be the most efficient way of further developing the student’s application of their knowledge of pathology in the clinical setting. This concept is supported by adult learning theory that “learning is effective and motivating when it is relevant to the solution of real-life problems” ( Kauffman and Mann, 2014).

### 2.2 Design

The teaching activity comprised the following steps:

The course was held over four sessions, each consisting of a digital image presented online followed by a two-hour-long face-to-face (f2f) tutorial session for all students held at fortnightly intervals.

Step 1: With an average student cohort of around 30 students they are usually divided into five teams of six students. All students had a briefing session when learning objectives were outlined and involved seven steps

1. Identify the specimen
2. Describe any abnormal features
3. State a possible diagnosis
4. Describe the likely symptom(s)
5. State any diagnostic tests
6. Give an outline of treatment(s)
7. Describe the possible preventative measure

**Figure 1:**
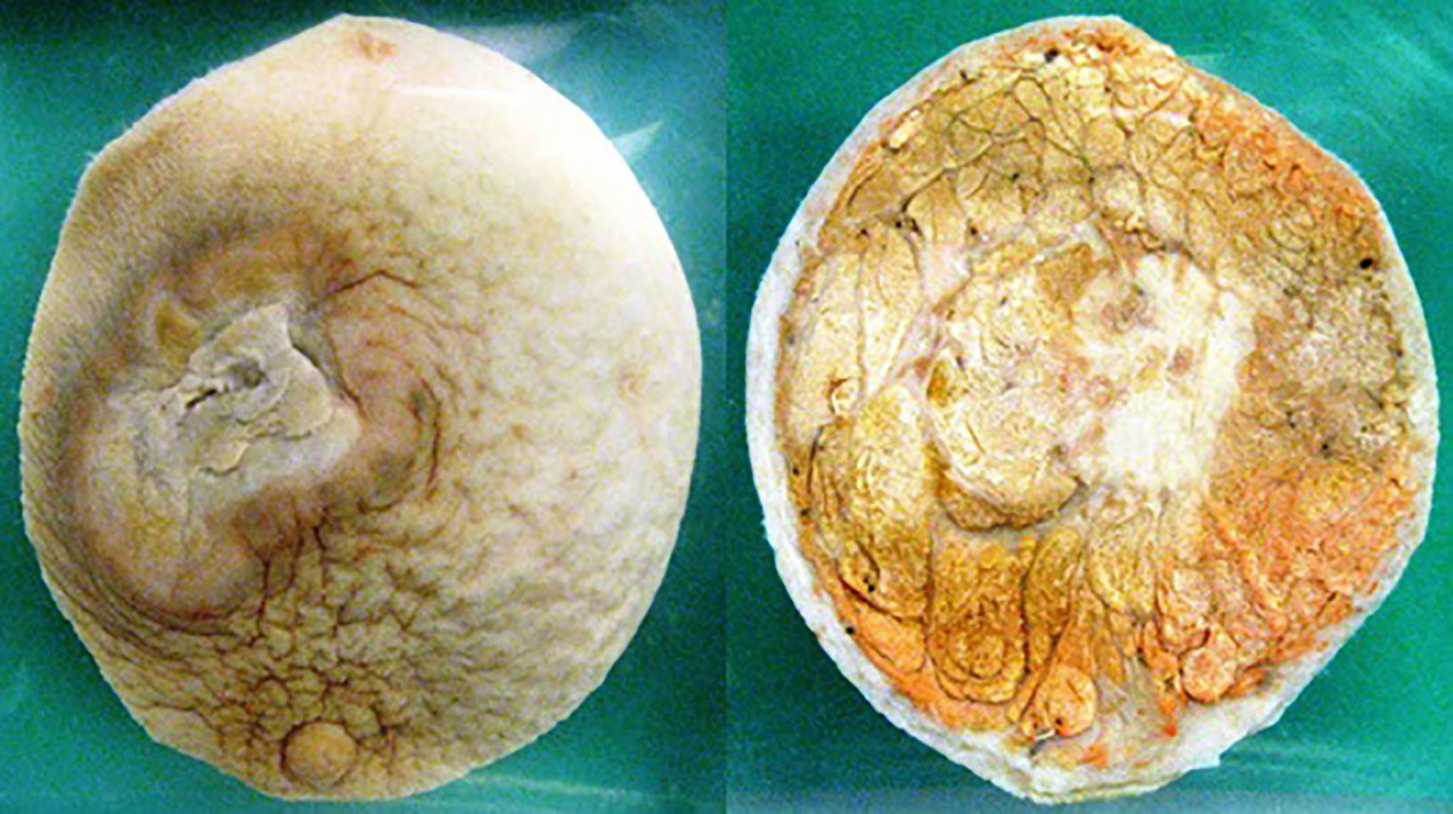
Example of a pathology ‘pot’ specimen

Each team was assigned an online digital image of a pathology specimen (‘pot’) (see Figure 1) placed on an online discussion board

The ‘pots’ are chosen from our existing pathology museum and based on the weekly topic for discussion e.g., upper gastrointestinal tract, the hepatobiliary system including pancreas, lower gastrointestinal tract, breast, and thyroid. Using the on-line discussion board one of the students from each team answers an initial question from the tutor. After answering the initial question, the student has to pose a question to another student in their team and this question and answer continue in rotation from student to student until all have attempted a question. This leads to a progressive accumulation of information gathered about the specimen and a response shown below -

Example of a student responding on the online discussion session with a reference.

“The patient may notice a lump in their breast, this can be a hard fibrous lump or a soft lump. Lumps like this can often appear under the arm or swelling appearing in the armpit. With this, they could also identify a change in the size or shape in their breast, this could be one or both breasts. Dimpling on the skin of the breast is also a common symptom of breast cancer as well as a change in the appearance of their nipple being more sunken into the breast tissue. As well as this some patients also notice a discharge from the nipple that can sometimes be streaked with blood. Pain in the breast or breast affected is also a fairly common symptom. Healthdirect.gov.au. (2019). *Breast cancer symptoms.* [online] Available at: https://www.healthdirect.gov.au/breast-cancer-symptoms [Accessed 1 Apr. 2019]”. (The link here is a reference used by the student). When the team discussion is complete, students are encouraged to read the responses of other teams and add further comments to the discussion. The online discussion was asynchronous, allowing flexibility for both students and tutor.

Step 2: Students were encouraged to undertake further research on answers and reflect on any issues raised by accessing additional resources or by contacting experts in the field. Any extra findings could be communicated to the tutor for dissemination to other groups or brought up at the next f2f session.

Step 3: Before the f2f session, held on alternate weeks, the tutor (author) reads the online discussions and checks for accuracy, and validity of evidence(s) offered. Discussions during the f2f sessions allow exploration and debate between teams on issues about diagnosis, diagnostic methods, treatment, preventive measures, and any inaccuracies or ambiguities within the answer sets. During the discussion, the students themselves are encouraged to provide most of the answers with the tutor’s role being ‘guide on the side’ rather than ‘sage on the stage’. It is important in this process to get the answer right, but also to ensure the students reflect on the *process of answering.* This involves the process of sifting through the possibilities of identification and diagnosis. It is often their first experience of inductive problem-solving.

Assessment of the program:

a. Formative assessment: this was conducted with multiple-choice questions (MCQs) based on the topics of the four tutorial sessions and was performed with 15 pre and post-course MCQs utilising TurningPoint ^TM.^
b. Summative assessment: this was started in 2008, by introducing a pathology specimen at one of the Objective Structured Clinical Examination (OSCE) stations, underpinning the pivotal role of pathology in patient management. To test the effectiveness of the OSCE station, scores were analysed from a group who had standard f2f learning only (before the introduction of the present format of blended learning) to compare with the scores from the group who had online and f2f format. However, the OSCE station was discarded later due to the observed lack of any difference between the groups and logistical difficulty in administering the pathology specimen in every OSCE.
c. Evaluation of the program was undertaken using a questionnaire (Likert scale 1 to 5) in the final week ( Table 2) with the opportunity to also provide open-ended comments. The voluntary nature of the assessment means that data gathering has been varied and in many cases, the numbers were too small to be statistically analysed.

Ethical approval was granted by the Tasmanian Human Research Ethics Committee (Reference number: H0015203) for the students’ voluntary participation in pre-and post-course assessments and evaluation of the program.

## Results/Analysis

From its initial conception, the course has consistently gained good feedback from students but the iterative nature of the course development has meant that over time some aspects of the course and evaluation process have changed. Changes and the voluntary nature of the evaluation meant limited response numbers within many cohorts that have impacted data capture and analysis. Given these constraints a cohort from 2015 has been chosen because it had the best number of pre/post pairs available for testing and analysis.

Results are for matched individual students in the pre and post-tests (N=21) and indicate the lowest individual minimum score in the pre-test was 27% correct answers which increased to 40% correct answers in the post-test, a 13% rise of correct answers. The highest individual maximum score across tests went from 80% to 87%, a change of 7%. The greatest individual improvement was 27%, and across students the average improvement was 10%. Two students (10%) had the same results across the two tests, three (14%) had a decrease in scores and sixteen (76%) students increased their scores in the final test. The positive change in test results for most students over the period of the course is encouraging and suggest that weaker students may gain real benefits from this teaching and learning format. Also, the number of students scoring less than 50% fell from six to three percent.

A paired-samples 2-tailed t-test was conducted to compare learning outcomes from the first-week pre-test to the final week post-test. There was a very significant difference in the scores for the pre-test quiz (M=54.24, SD=13.23) and the post-test quiz (M=64.43, SD=14.26); with *t*(21)= -4.425, *p* = .000 and suggests the learning process was effective. Specifically, the results suggest that when students view pathology specimens online, engage in scholarly debate about them, and participate in face-to-face workshops to consolidate their learning, they develop strong inductive medical reasoning skills (see also discussion on E5 and E6 below).

In 2020 there are only post-test results available and analysis of rotations indicates the students during the Covid period have slightly improved over the year and overall did slightly better than the 2015 cohort ( Table 1).

**Table 1:** Comparison of results for 2015 and 2020

	2015 Rotation	2020 Rotation (a)	2020 Rotation (b)	2020 Rotation (c)
Highest score	87%	87%	93%	100%
Lowest score	40%	47%	47%	47%
Average score	64%	62%	68%	72%
S.D.	14.26	10.65	14.66	14.25
N	21	27	25	28

**Table 2:** Results of the level of satisfaction for one cohort of 2015 students (N=24)

	Strongly agree/Agree	Neutral	Strongly disagree/Disagree
E1: I found learning with combined online and face to face sessions very useful	90%	7%	3%
E2: Download times of teaching materials were satisfactory	83%	12%	5%
E3: The content of the course was adequate within the timeframe	95%	5%	0%
E4: The on-line sessions have helped me to feel like an independent learner	88% (2020 - 81%)	5%	7%
E5: In this course, I have learned to think critically	83%	15%	2%
E6: learning in these sessions helped me to connect radiological and pathological processes of disease	98%	2%	0%
E7: I have learned to apply evidence-based medicine to the management of patients	85%	13%	2%

Table 2 indicates the level of satisfaction for all students (includes those who could not be matched across tests N=24) from the 2015 cohort and indicates the teaching process is highly valued with an average of 89% Strongly Agree/Agree across all 7 items used in the evaluation and comments reaffirm this: *(2015 comments “It was good to follow the online path with a face-to-face tutorial”; “I have very much appreciated these sessions”).* The Strongly agree/Agree responses to items E5 and E6, 83% and 98% respectively, reflect the students’ positive perceptions about their critical thinking and ability to apply their pathology knowledge in clinical situations.

The evaluation questions were altered in 2020 to reflect the changed teaching environment. The only question directly comparable across the two-time frames is E4 and the 2020 result (in brackets) is similar to the 2015 result as shown in Table 2. Comments also indicate the students appreciate the course even with Covid restrictions ( *2020 comment “I feel the way in which you guide us to the answer to your questions, instead of giving us the answers, to be very effective in helping us better understand and remember important concepts.”)* as well astheir view on critical thinking *(2020 comment “Your way of explaining concepts by building up from the basics and getting us to think through them is very effective, and I feel like I have a much better approach to tackling unfamiliar problems now.”*)

A comparison of scores obtained from the OSCE station on pathology specimens between the groups who only had f2f learning with the group who had both online and f2f revealed no significant difference between these two cohorts with regards to examination scores respectively (SD-2.41, mean-14.67 and SD-2.83, mean-13.68).

## Discussion

The teaching of surgical pathology by a surgeon is not new. It is well known that Sir James Paget, at the St Bartholomew’s Hospital in London ‘held students captive with his lectures on surgical pathology’ and also at the London Hospital Medical College, where surgeons gave lectures in surgical pathology ( Domizio, 2006). The strategy in this program development was to deliver pathology education to medical students that integrated prior basic science knowledge into clinical learning. This resonates with the statement by the surgeon-anatomist, Professor Harold Ellis, “...99% of medical students are really only interested in the ‘customer’ - the living, breathing patient. Unless the clinical situation is kept at the forefront, then theory seems not only dull but also a waste of time.... So, the ideal situation is, of course, to bring clinicians and pathologists, as well as related specialists, together in order to teach.” ( Ellis, 1991)

The course involved students’ developing evidence-based reasoning skills towards the identification, diagnosis, and treatment of conditions observed in pathology specimens. ( *2015 comment “Presentation + questions complemented by pots very useful.”; 2020 comment “* *Some of the path specimens were a bit challenging to identify online, but through some group discussion they were not too difficult to figure out! I felt like I learned a lot through these sessions”)* *.* In 2012, Kenwright at the University of Otago, Wellington, in a conference abstract mentioned using combined online learning with face-to-face teaching and embedding the material within a clinical block ( Kenwright, 2012).

The present study demonstrates that a surgeon and clinicians (some from other specialties) can contribute towards students learning of pathology utilising both blended learning and the TPACK frameworkfor a better understanding of the process of patient management ( Koehler and Mishra, 2008).

Using online discussions, and in groups, the students were required to identify the organs using the online images based on their anatomical knowledge. Going further, they also had to describe the pathology and think about possible clinical signs and symptoms to arrive at a clinical diagnosis ( *2015 comment “The on-line session has helped me to feel I was an independent learner”; 2020 comment* *“I feel the way in which you guide us to the answer to your questions, instead of giving us the answers, to be very effective in helping us better understand and remember important concepts”)* *.* This addresses the first research question as the pedagogical model requires students to develop their observational understanding of pathophysiology. In this process, students develop their clinical skills and ability to ‘see’ the patient around the specimen. In this context, the object becomes not just a ‘pot’ but a part of a body and the consideration of possible clinical signs and symptoms to arrive at a clinical diagnosis. In developing a diagnosis they consider, and justify, their findings and pass this information on to another student with a subsequent question to answer. This reverses the traditional ‘clinicopathological conference’ model, where the clinician attempts to reach a diagnosis based on clinical history, examination, and investigations followed by the pathologist confirming the final diagnosis. ( *2015 comment “I enjoyed learning how to approach a case with critical thinking and how to question and justify the assumptions and thinking myself and [to] others”; 2020 comment* *“Having a chance to check our knowledge is a very rare thing in this course and any opportunity is invaluable”).*

In addressing the second research question, although this model does not simulate clinical practice where the clinician is presented with a full patient, not just a diseased organ, by visualizing the patient with the diseased organ diagnostic considerations are developed through the association of symptoms and signs with the observed pathology. Thus “...the subject of pathology in all fields (would only) become alive when related to reality...” ( Ellis, 1991). This new teaching model fosters ‘backward thinking’ (inductive approach) as opposed to the traditional ‘clinicopathological conference’ model (deductive approach). ( *2015 comment “Interesting topics and stimulated our thinking.”;* *2020 comment* *I really liked how you talked through the initial bit of each case and let students present their part of the case”).*

In Roman mythology, the god Janus had many tasks, one of which was as the gatekeeper of heaven and the guardian of gates by looking in two directions, east, and west. As such, he was commonly represented as having a head with two faces - one facing forwards and the other backward ( Wasson, 2015).

Apt here is another aspect of Janus as he represents change and transition with a face to the past and the other to the future. In this present study, the past is represented by the specimens to predict a future - the trajectory of the disease within the patient, diagnosis, treatment, and hopefully recovery.

The decision-making process involves two different kinds of thinking: looking backward to understand the past and looking forward to predicting the future. In clinical practice, inductive diagnostic reasoning skills require clinicians to *think about how they think.* Success in this metacognition and inferential thinking depends on skills of diagnostic reasoning with a deep understanding of clinical manifestations arising from the underlying pathology. This program aids and builds constructive alignment and knowledge construction through clinical reasoning, allowing the development of skills in diagnosing and managing diseases during the students’ clinical practice.

There are limitations within this study such as the absence of a control group for the pre and post-test quiz, data from only two years, and the teaching only being delivered by a single tutor (surgeon). In addition, as the participation in the quiz was optional, the data gathered was limited and presented only from a group with reasonable numbers of students who participated in both pre and post quizzes. The University Teaching awards to the author for course development and the positive experience engendered in this course manifested by supportive comments from evaluations of the program over a long period may be seen to counterbalance these limitations. ( *2015 comment “The sessions were valuable in revising pathology content”; 2020 comment “The sessions were a very useful learning activity”].*

## Conclusion

With minor calls on medical school resources, the benefits of this blended learning program include the novel presentation of specimens using multimedia within a combination of learning situations - especially the online discussion which stimulates thinking proactively.

Self-development requires students to be open to change and to challenge themselves in critical thinking and medical reasoning by utilising the pathological process of diseases. This blended learning model encourages and develops medical knowledge, conceptual thinking, and disease management choices. It also fosters other important professional attributes such as teamwork within a group setting and the ability to communicate conclusions to peers and other health professionals both in writing (online) and verbally (f2f discussion).

In summary, this study provides supportive evidence that overall students’ pathology knowledge improved through delivery using blended learning and more importantly weaker students benefitted considerably. Also, the results suggest that when students view pathology specimens online, engage in scholarly debate about them, and participate in face-to-face sessions to consolidate their learning, they gain strong inductive medical reasoning skills. The results and comments from the rotations in the 2020 intake also allude to a continuity in student outcomes within the course even allowing for different Covid related teaching and tutorial arrangements *(2020 comment* *“Thanks, this was a good learning experience I found the tutorials to be a valuable learning experience, and a good adaptation of what is supposed to be an in-person learning opportunity”).* The results presented in Table 1 suggest that the course teaching methodology, student involvement, and test outcomes are consistent and robust over time and working well in a Covid restricted teaching environment and comments are also consistent *(2020 comment* *“I think the module was extremely well presented and was very flexible for me as a student, with the ability to complete tasks at my own pace prior to sessions and review additional material as necessary”).*

Returning to Janus - medicine itself is rapidly changing driven in large measure by technological advances. Medical education can also embrace beneficial change by using technology constructively especially as we enter a post-Covid world.

Finally, the Covid pandemic crisis has put unexpected pressure on many systems including the medical system which includes, amongst many others, teaching a new generation of doctors. An unexpected bonus of this style of teaching is its flexibility and adaptability to the new restrictions. *(2020 comments “* *Thank you so much for adapting so well to teaching us online”; “This way of delivery was good considering the circumstances”; “I have struggled with the switch to online learning however the delivery of these tutorials has been great, and the format is excellent!”).*

Given major changes occurring in a Covid era teaching environment and with just minimal changes to the f2f meetings, the students were still able to, and engage in, learning about pathology with little impact on knowledge transfer *(2020 comments* *“I had an overall very positive experience with the online learning”; “I have really enjoyed the tutorials and found them very helpful (especially because pathology is not my strong suit!”).*

In essence, the described model provides a flexible and innovative process that involves students in the learning process and where clinicians can contribute, enhance, and add to existing pathology learning for medical students within a clinical context.

## Take Home Messages

• Studying pathology from diseased organs help students to learn critical thinking and clinical reasoning skills.
• Students can develop a better understanding of the pathological process and its relationship with clinical symptoms and signs through supplementary pathology teaching by a clinician.
• The model adds to existing pathology learning for medical students within a clinical context.
• This study provides supportive evidence that overall students’ pathology knowledge improved and more importantly weaker students benefitted considerably.
• This model fosters teamwork and ability to communicate to peers and other health professionals both in writing (online) and verbally (f2f discussion).

## Notes On Contributors

**Professor Sinha** has an extensive background in medical education and received the Medal of the Order of Australia for his outstanding contributions to medical education and wound care in 2005 and University of Tasmania Vice-Chancellor’s Medal for Sustained Commitment to Teaching Excellence in 2019. He was a general surgeon practicing in Australia, India, Zambia and Papua New Guinea. He is currently a Professor in Surgery (Wound Care) at the University of Tasmania and Professor and Head of Anatomy at the University of Notre Dame Australia Sydney. Professor Sinha - ORCiD: https://orcid.org/0000-0002-9676-4480
