# Supplementary material for: Enhancing pathology learning for medical students – via blended learning by clinicians
Source: MedEdPublish (2016). 2021 Mar 29;10:82. [Version 1] doi: 10.15694/mep.2021.000082.1 (PMC10939644; doi:10.15694/mep.2021.000082.1)
Supplement: Peer review history

# Reviewer report: Reviewer response for version 1

**DOI:** 10.21956/mep.19090.r27503

**Published:** 2021-06-03

**Authors:** Michael SH Wan

**Affiliations:** 1 University of Notre Dame

This review has been migrated. The reviewer awarded 4 stars out of 5 Congratulation to the author in developing a blended learning module to enhance students' learning in anatomical pathology with clinical relevance. The detailed description of the design and implementation in the methods section could be adapted by other medical schools. Although the program is mainly conducted by one clinician, the expansion to a team teaching approach in the future would further enhance the teaching and learning of this discipline for medical students.

Reviewer Expertise:

NA

No decision status is available

# Reviewer report: Reviewer response for version 1

**DOI:** 10.21956/mep.19090.r27500

**Published:** 2021-04-13

**Authors:** Rajaei Sharma

**Affiliations:** 1 College of Medicine and Health, University of Exeter

This review has been migrated. The reviewer awarded 3 stars out of 5 This is an interesting paper that addresses an important aspect of medical education – one that, in recent times, seems to have in fact lacked attention due to the widely accepted status quos. In an era where so much effort and attention is being directed at online and virtual resource, this is a timely reminder of the importance of pathology and face-to-face teaching.The introduction provides a very well written and wide-ranging background as to the changes seen in medical education, pathology teaching and the effects that can be seen beyond graduation. The methods then nicely details the more salient points and provides all of the necessary specifics that the reader would need. Although the author does describe how the program has been developing since 2008, it would be appreciated if some of the references were more up to date. If not possible, this would then provide an opportunity for a comparison to be made with more recent literature and would indeed strengthen the authors statements of how education has changed. The style and format of the paper has been well thought out with a nice blend of commentary style writing and more concise statement sections. Figures and tables have been used in a purely additive fashion and there is no unnecessary imagery. In the analysis, it may be useful to provide further comments as they were likely a large factor of the feedback. These could be included as an image for clarity. Throughout the paper there is a statement that the weaker students may benefit the most, however from the way in which the results are described, the origin of this statement is not abundantly clear. As it is one of the more key conclusions, it would be useful to further clarify how this was determined within the results.Due to the aforementioned changes that have been seen recently in medical education, it may be pertinent to include (or separately write about) any further results that can be drawn from the data comparing F2F learning vs online only as mentioned at the end of the analysis section. The discussion is well written and draws on the most important results seen whilst also being aware of the limitations of the data.The points listed here are additive in nature and do not highlight any concerns with the paper. The most important point to address would be clarity over the conclusion regarding weaker students. Overall, this is a well written paper and research that is continually important to the continuing development of medical education globally.

Reviewer Expertise:

NA

No decision status is available

# Reviewer report: Reviewer response for version 1

**DOI:** 10.21956/mep.19090.r27499

**Published:** 2021-04-04

**Authors:** Mary Higgins

**Affiliations:** 1 University College Dublin, National Maternity Hospital

This review has been migrated. The reviewer awarded 3 stars out of 5 Thank you for this beautifully written paper. You acknowledge yourself that a limitation of this is that the programme was given by one clinician, and one that has obviously significant clinical experience as well as national recognition for excellence in education. Can you postulate if this programme would be as successful if another gave it? Minor point: for statistics, my understanding was that n (representing a sample) is preferable to N (representing the entire population that can be sampled)

Reviewer Expertise:

NA

No decision status is available

# Reviewer report: Reviewer response for version 1

**DOI:** 10.21956/mep.19090.r27501

**Published:** 2021-03-30

**Authors:** Simonetta Ausoni

**Affiliations:** 1 Department of Biomedical Sciences- University of Padova- School of Medicine

This review has been migrated. The reviewer awarded 4 stars out of 5 I have read with great pleasure the paper by Sankar Sinha. The study described is well finalized and full of ideas that can be easily translated in different settings in the medical courses. Sinha proposes to utilize images of pathological specimens as tools to link organ and disease identification with clinical reasoning and diseases diagnosis. Important aspects of this methodology are: 1. The use of images instead of true diseased organs, a procedure that can be easily applied both in a face-to-face session and online; 2) The student-centered methodology applied in each session, with a continuous rotation from student to student, to generate questions and answers and reach the final diagnosis; 3) The possibility to develop an inductive medical reasoning approach, starting from early years of medical courses. Survey analysis presented in this study indicate that the approach improves knowledge in pathology, the diagnostic capacity and the clinical reasoning. My only concern is that it requires a well-organized repository of images to allow a reasonable case rotation. Anyway, an interesting model to adopt!

Reviewer Expertise:

NA

No decision status is available

# Reviewer report: Reviewer response for version 1

**DOI:** 10.21956/mep.19090.r27502

**Published:** 2021-03-30

**Authors:** Balakrishnan Nair

**Affiliations:** 1 Centre for Medical Professional Development and University of Newcastle

This review has been migrated. The reviewer awarded 4 stars out of 5 I enjoyed reading this article. Pathology is an essential part of medical learning, and often it is taught in isolation. So this paper to show that we can teach pathology in context is a welcome change. Students get excited and learn better when taught in this way. The study has other essential elements such as peer-assisted learning, self-directed learning and critical thinking.The evaluation has shown that it helps weaker students and the learners well accepted the program.

Reviewer Expertise:

NA

No decision status is available
